# Steel Wire Rope Surface Defect Detection Based on Segmentation Template and Spatiotemporal Gray Sample Set

**DOI:** 10.3390/s21165401

**Published:** 2021-08-10

**Authors:** Guoyong Zhang, Zhaohui Tang, Ying Fan, Jinping Liu, Hadi Jahanshahi, Ayman A. Aly

**Affiliations:** 1 School of Computer Science and Engineering, Central South University, Changsha 410083, China; csu_zgy@csu.edu.cn; 2 School of Automation, Central South University, Changsha 410083, China; zhtang@csu.edu.cn; 3 Hunan Provincial Key Laboratory of Intelligent Computing and Language Information Processing, Hunan Normal University, Changsha 410081, China; ljp@hunnu.edu.cn; 4 Hunan Xiangjiang Artificial Intelligence Academy, Changsha 410081, China; 5 Department of Mechanical Engineering, University of Manitoba, Winnipeg, MB R3T 5V6, Canada; hadi_jahanshahi@ut.ac.ir; 6 Department of Mechanical Engineering, College of Engineering, Taif University, P.O. Box 11099, Taif 21944, Saudi Arabia; aymanaly@tu.edu.sa

**Keywords:** surface defect detection, segmentation template, spatiotemporal gray sample set, machine vision, sealed wire rope

## Abstract

Machine-vision-based defect detection, instead of manual visual inspection, is becoming increasingly popular. In practice, images of the upper surface of cableway load sealing steel wire ropes are seriously affected by complex environments, including factors such as lubricants, adhering dust, natural light, reflections from metal or oil stains, and lack of defect samples. This makes it difficult to directly use traditional threshold-segmentation-based or supervised machine-learning-based defect detection methods for wire rope strand segmentation and fracture defect detection. In this study, we proposed a segmentation-template-based rope strand segmentation method with high detection accuracy, insensitivity to light, and insensitivity to oil stain interference. The method used the structural characteristics of steel wire rope to create a steel wire rope segmentation template, the best coincidence position of the steel wire rope segmentation template on the real-time edge image was obtained through multiple translations, and the steel wire rope strands were segmented. Aiming at the problem of steel wire rope fracture defect detection, inspired by the idea of dynamic background modeling, a steel wire rope surface defect detection method based on a steel wire rope segmentation template and a timely spatial gray sample set was proposed. The spatiotemporal gray sample set of each pixel in the image was designed by using the gray similarity of the same position in the time domain and the gray similarity of pixel neighborhood in the space domain, the dynamic gray background of wire rope surface image was constructed to realize the detection of wire rope surface defects. The method proposed in this paper was tested on the image set of Z-type double-layer load sealing steel wire rope of mine ropeway, and compared with the classic dynamic background modeling methods such as VIBE, KNN, and MOG2. The results show that the purposed method is more accurate, more effective, and has strong adaptability to complex environments.

## 1. Introduction

**Figure 1:**
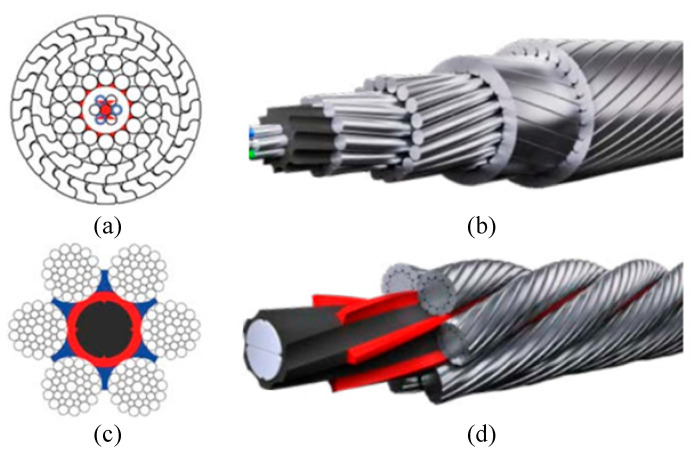
Main types and structure of wire rope. (**a**) Section of sealing wire rope; (**b**) Appearance of sealing wire rope; (**c**) Section of unsealed wire rope; (**d**) Appearance of unsealed wire rope.

In the field of surface defect detection, traditional manual inspection has the disadvantages of low accuracy, poor real-time defect detection, low efficiency, and high labor intensity. Machine-vision-based methods have the advantages of non-contact, high real-time, and no manual participation [1], these methods are increasingly being applied in modern industries [2,3,4,5,6]. In this study, we investigated machine-vision-based methods for detecting defects on the surface of steel wire ropes, using sealed wire ropes as the research object. Sealed wire ropes are widely used in aerial ropeway systems in mines or at scenic spots for transporting goods or carrying people. This type of wire rope is usually twisted from one to three layers of shaped steel wire wrapped with multiple strands of round heart steel wire, with a tight structure, a smooth surface, no rotation, and good sealing characteristics, with the section structure and appearance shown in Figure 1a,b. The sealed wire rope surface defects occur mainly because the wire rope is pulled apart by the pressure of the surface rope strand fracture to form fracture defects. As a result of the force exerted by the weight of goods and people, local broken wire develops into a concentrated broken strand in a short period. When the concentrated broken strand exceeds the scrap standard, the whole rope needs to be replaced, leading to a short life cycle. At the same time, in this short life cycle, the quantity of fracture defect data is small, which makes it difficult to use supervised learning methods to detect defects [7].

There is little literature on machine-vision-based defect detection for sealed wire ropes, but researchers have carried out a lot of work on surface defect detection for non-sealed ordinary wire ropes. A non-sealed wire rope plays an important role in lifting and traction systems. The difference between sealed wire rope and non-sealed wire rope lies in the composition and twisting method of wire rope. The main performance is that the appearance of non-sealed wire rope has finer texture than that of sealed wire rope, and these textures are more prone to wear, broken wire, corrosion, and other defects. The section structure and appearance are shown in Figure 1c,d.

Zhou et al. [8,9,10,11] studied in-depth non-sealed wire ropes and proposed a wear detection method based on a deep convolutional neural network that involved training a large number of labeled wear sample images to detect the wear degree of wire ropes. To address the influence of light noise, using texture features, the uniform local binary pattern operator (u-LBP) and principal component analysis methods were proposed to detect the wire rope defect area. In addition, to solve the wire rope surface defect classification problem, an optimized support vector machine method based on uniform local binary features and grayscale co-occurrence moment features was proposed. Xh et al. [12] proposed a convolutional-neural-network-based intelligent method for detecting wire rope damage by training a model with a large number of damage sample images. Shi et al. [13] used the infrared measurement method to determine wire rope wear using the Canny edge detection method with an infrared camera. From the perspective of structural modeling, Wacker et al. [14] proposed a combination of structure- and appearance-enhanced wire rope anomaly detection to design probabilistic appearance models to detect defects. To achieve better-supervised learning of defect models, Platzer et al. [15] proposed a new strategy for wire rope defect localization using hidden Markov models. To solve the problem of wire rope twist distance measurement, Vallan et al. [16] established a mathematical model of a wire rope profile and a vision-based twist distance measurement technique for a metal wire rope. Dong et al. [17] detected wire rope defects by extracting texture features such as the smoothness and entropy of the wire rope surface. Ho et al. [18] combined image enhancement techniques with principal component analysis (PCA) for detecting wire rope surface defects. The above methods have achieved good results in the surface defect detection of unsealed steel wire rope, but they depend on a large amount of defect data and are not suitable for the defect detection of sealed steel wire rope.

**Figure 2:**
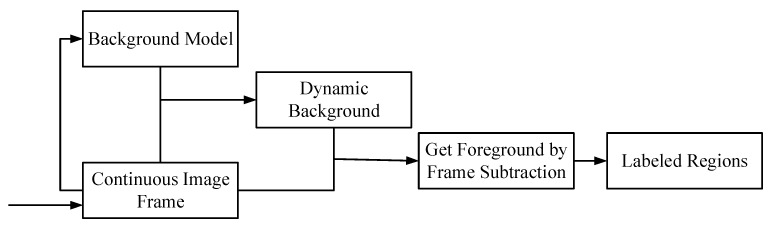
The process of background modeling.

When the outdoor camera is fixed, the changing area in the fixed scene is extracted, and the dynamic background modeling method is usually used. The background modeling process is shown in Figure 2. The background model is dynamically constructed for the real-time image sequence, and the scene change area, that is, the foreground area, is obtained through the difference between the dynamic background and the real-time image frame. For the continuously collected outdoor sealed steel wire rope images, the change of pixel gray in the surface area of steel wire rope is similar to the application scene of the background modeling method. In the time domain, the pixels in the image sequence of wire rope surface area at the same position may be rope strand edge, rope strand surface, oil sludge, or fracture, but the gray levels of pixels in one type are similar in the time domain. At the same time, the gray levels of rope strand edge and fracture are similar, the gray levels of wire rope surface and oil sludge are similar, but the gap gray level is significantly different from that of wire rope surface; In the spatial domain, the gray level of the rope strand area pixels or the rope strand edge area pixels in the wire rope surface area image is similar. According to the above wire rope image characterization, the change model of a single-pixel in the image can be established, and the defect area on the wire rope surface can be extracted by using a methodology similar to dynamic background modeling. The classical dynamic background modeling methods such as the visual background extractor (VIBE) method proposed by O. Barnich et al. [19,20], which established a random replacement background model for each pixel; an adaptive background mixing model based on KNN (k-nearest neighbor) proposed by Stauffer C et al. [21], which modeled each pixel and realized foreground segmentation by using the idea of nonparametric probability density estimation and KNN classification; the MOG2 method of Gaussian mixture model proposed by Zivkovic Z [21,22,23], which realized dynamic background modeling by using parametric probability density estimation, Gaussian mixture distribution, and shadow detection [24].

**Figure 3:**
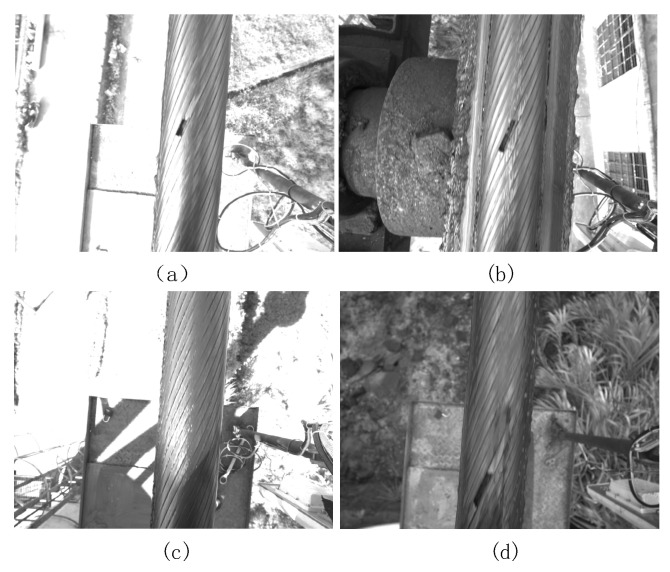
Complex representation of wire rope image. (**a**) Strong light and reflection; (**b**) Weak light; (**c**) Uneven light; (**d**) Oil stain on wire rope surface.

The sealed steel wire rope of freight ropeway studied in this paper is applied to the outdoor environment. The collected surface images of steel wire rope are difficult to distinguish defects due to the following reasons: (1) the edge of the rope strand is blurred due to strong light, weak light, reflection, uneven illumination, and shadow, as shown in Figure 3a–c; (2) It is difficult to distinguish the edge of the rope strand due to oil stain on the surface, as shown in Figure 3d; (3) The surface texture of steel wire rope changes irregularly along the motion direction between adjacent image frames. The above characteristics make it difficult for the existing dynamic background modeling methods to effectively segment the strand on the surface of steel wire rope and detect fracture defects.

**Figure 4:**
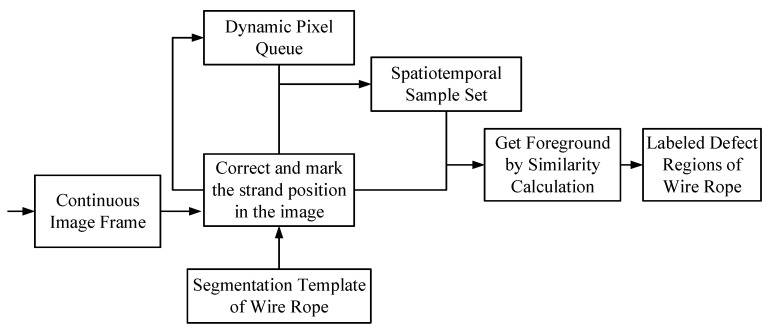
The main technical route of the proposed method.

In order to solve the above problems, this paper proposes a steel wire rope surface defect detection method based on the segmentation template and the spatiotemporal gray sample set. The technical route of the method is shown in Figure 4. The steel wire rope segmentation template is constructed in advance, the rope strands of the real-time steel wire rope image are segmented and corrected by using the template, and the pixel information is extracted from continuous multi-frame images to build a dynamic pixel queue, combined with the rope strand information, the spatiotemporal gray sample set is constructed, the similarity between the real-time image and the spatiotemporal gray sample set is compared, and the defect area in the steel rope surface image is extracted and marked. The main contributions of this paper are as follows:

(1) Based on the geometric and texture features of the sealing wire rope, the segmentation template of the sealing wire rope is created for the first time;
(2) The strand segmentation and correction method based on the steel wire rope segmentation template is proposed, which effectively solves the problem of strand segmentation caused by the variable speed movement and vibration of the ore hopper car;
(3) The spatiotemporal gray sample set of pixel points in the relative motion environment between the camera and the scene is constructed for the first time and is used for wire rope defect detection, which effectively solves the problem of defect detection caused by light and oil pollution.

The remainder of this paper is organized as follows: a steel wire rope surface defect detection method based on steel wire rope segmentation template and spatiotemporal gray sample set is proposed in Section 2. Section 3 describes the content of algorithm implementation. In Section 4 and Section 5, the validity of the method is verified experimentally and the conclusions of this study are given.

## 2. Steel Wire Rope Surface Defect Detection Method Based on Segmentation Template and Spatiotemporal Gray Sample Set

### 2.1. Creation of Wire Rope Segmentation Template

Rope strand information is one of the bases for judging the surface defects of steel wire rope. The fracture defects connect at least one pair of adjacent rope strand edges. When the camera is fixed on the bucket car and moves along the rope direction, according to the structural characteristics of the sealing wire rope, the texture law of the strand edge in the wire rope surface image is summarized, and the sealing wire rope segmentation template is constructed. The template is a priori knowledge of strand segmentation and defect detection. Each new steel wire rope creates a steel wire rope segmentation template before usage, which only needs to be created once in the service life cycle of the steel wire rope.

**Figure 5:**
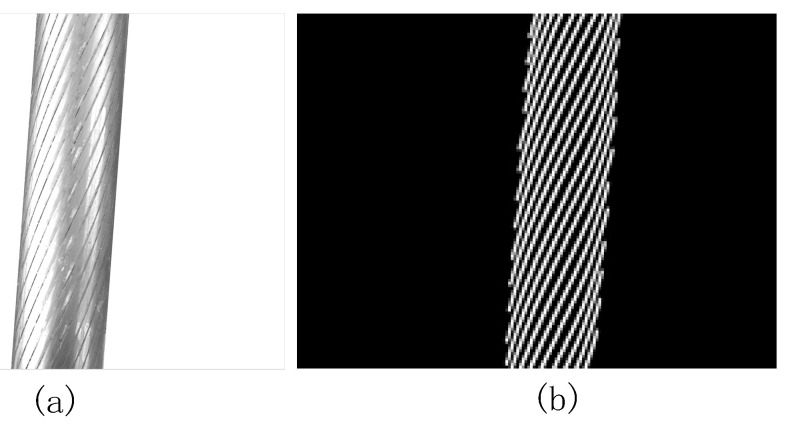
Segmentation template for wire rope. (**a**) Wire rope image; (**b**) Segmentation template.

In the preparation stage, in order to create a steel wire rope segmentation template, wipe the oil stain on the surface of the steel wire rope until the texture is clear, add a white background plate, and collect a steel wire rope image; the line is detected by Hough transform to obtain the left boundary $y_{l}$ and the right boundary $y_{r}$ of the steel wire rope, given by:

(1) $$y_{l}=ax+b_{1}$$

(2) $$y_{r}=ax+b_{2}$$

where $0\leq x\leq w,\;0\leq y_{l}\leq h$, $0\leq y_{r}\leq h$, w is the image width, h is the image height, and $a,b_{1},b_{2}$ are linear formula coefficients. OSTU (Nobuyuki Otsu Method) [25] adaptive threshold segmentation is performed on the collected image. The gray value of darker pixels is set to 1 and the gray value of brighter pixels is set to 0. The connected domain of the resulting image is analyzed and the connected domain with smaller areas is removed. Finally, the marked steel wire rope segmentation template $I_{SEG}\left(x,y\right)$ is obtained. Figure 5a is the steel wire rope image and Figure 5b is the segmentation template.

### 2.2. Strand Segmentation and Correction Based on Wire Rope Segmentation Template

**Figure 6:**
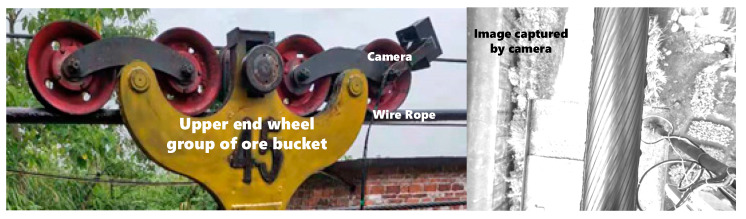
The environment of wire rope image acquisition.

In practical application, the image acquisition equipment is erected on the axle of the ore bucket car. As shown in Figure 6, the image acquisition equipment is in relative motion with the steel wire rope, the image of the upper surface of the wire rope collected by the MV-CA030-10GC industrial camera (Hikvision, Hangzhou, China). The left and right positions of the rope in the real-time steel wire image collected by the equipment are basically consistent. Although the surface texture of the steel wire rope changes rapidly along the rope direction, the steel wire rope segmentation template can be translated a certain distance along the rope direction to make the edge information of the rope strand in the template coincide with the edge information in the real-time image. Based on the above characteristics, in order to solve the problem of rope strand segmentation under the condition of oil sludge coverage, reflection, and uneven illumination, a rope strand segmentation and correction method based on the steel wire rope segmentation template is proposed in this paper.

The method is based on edge information. The rope strand edge texture in the image is seriously affected by oil sludge, uneven illumination, and reflection, and the rope strand edge information is incomplete. In order to solve the problem of edge extraction of wire rope surface image affected by noise, the FoGDbED method proposed by Zhang G et al. [26] was used to extract edge pixels in the image, which could effectively extract edges from the image affected by noise. The edge image C(x,y) of the current image is given by:

(3) $$C\left(x,y\right)=f_{FoGD}\left(I\left(x,y\right)\right)$$

where I(x,y) is the current image, $f_{FoGD}()$ is edge detection function of the FoGDbED method.

**Figure 7:**
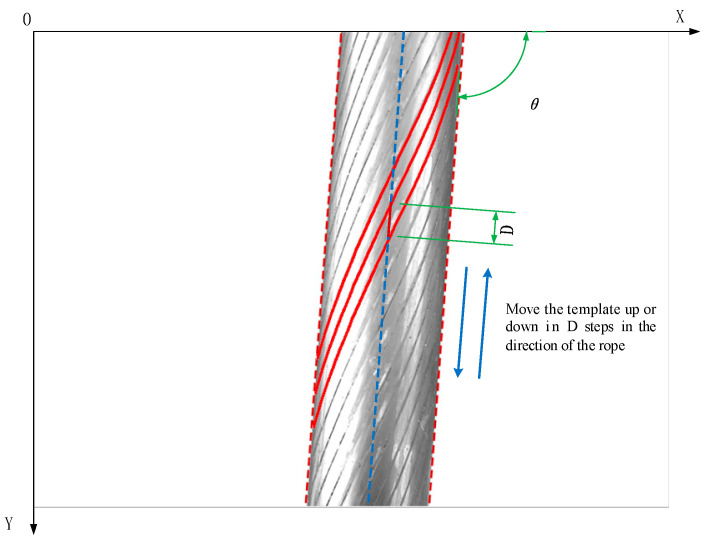
Explanation of shift operation.

The method proposed in this paper uses the steel wire rope segmentation template to translate in the rope extension direction, match with the edge information, calculate the coincidence degree response value, and select the corresponding position of the maximum coincidence degree response value as the best matching position. Where the translation operation can be seen as a translation calculation using the vector (dx,dy) for the wire rope segmentation template $I_{SEG}\left(x,y\right)$;

(4) $$M\left(x,y\right)=I_{SEG}\left(x+dx,y+dy\right)$$

where $I_{SEG}\left(x,y\right)$ is the steel wire rope segmentation template and M(x,y) is the translated image. The translation operation is carried out along the extension direction of the rope. The translation angle can be calculated from the slope a in Formula (1). The single translation vector (dx,dy) satisfies the constraint:

(5) $$\{\begin{array}{c} \sqrt{dx^{2}+dy^{2}}=1\\ \frac{dy}{dx}=a \end{array}$$

where a is the slope of $y_{l}$ or $y_{r}$. Each translation distance is the downward movement distance 1 along the rope direction. The farthest translation distance of the translation operation satisfies the constraint:

(6) $$\overset{n}{\underset{i=0}{\sum }}\sqrt{dx_{i}^{2}+dy_{i}^{2}}\leq D$$

where n is the number of times of translation, and the final translation distance is less than the gap distance D between adjacent strands in the extension direction of the steel wire rope, as shown in Figure 7. The reason is that the steel wire rope division template must coincide with the gap between the steel wire rope strands within the translation distance D in the extension direction of the steel wire rope, as shown in the figure, θ is the angle of steel wire rope, satisfied a=tanθ.

After the *j*-th translation operation of the wire rope segmentation template, the calculation rule of coincidence degree response value $R_{j}$ between image $M_{j}\left(x,y\right)$ and real-time image edge extraction result C(x,y) is as follows:

(7) $$S\left(x,y\right)=\{\begin{array}{l} 1,\;M\left(x,y\right)>0\;and\;C\left(x,y\right)>0\\ 0,\;otherwise \end{array}$$

(8) $$R_{j}=\overset{w}{\underset{x=1}{\sum }}\overset{h}{\underset{y=1}{\sum }}S\left(x,y\right)$$

where S(x,y) is the matching value between the wire rope segmentation template M(x,y) and the edge image C(x,y) of the real-time image after the j-th displacement at the coordinate (x,y).

When the steel wire rope segmentation template is known, the specific implementation process of the rope strand segmentation and correction method (RSCM) based on the steel wire rope segmentation template is shown in Algorithm 1.
**Algorithm 1.** Rope-strand segmentation and correction method based on steel wire rope segmentation template.**Inputs:** wire rope image R(x,y), steel wire rope segmentation template $I_{SEG}\left(x,y\right)$ **Output:** N(x,y) with strand edge markers 1: detect R(x,y)’s edge with FoGDbED detector and get edge image C(x,y) 2: $d=1,\;R_{max}=0,\;max=1$ 3: detect edge image C(x,y) with FoGDbED detector 4: $I_{SEG}\left(x,y\right)$ Shift distance 1 to get $M_{1}\left(x,y\right)$ with vector (dx,dy) 5: get $S_{1}\left(x,y\right)$ with Formula (7) and get $R_{1}$ with Formula (8) 6: $R_{max}=R_{1},\;max=1$ 7: **while** d≤D 8:   d++; 9:   I(x,y) Shift distance d to get $M_{j}\left(x,y\right)$ with vector (dx,dy) 10:   get $S_{j}\left(x,y\right)$ with Formula (7) and get $R_{j}$ with Formula (8) 11:  **if** $R_{max}<R_{j}$ 12:    $R_{max}=R_{j},max=d$ 13:   **end if** 14: **end while** 15: $N\left(x,y\right)=M_{max}\left(x,y\right)$ 16: return N(x,y)

**Figure 8:**
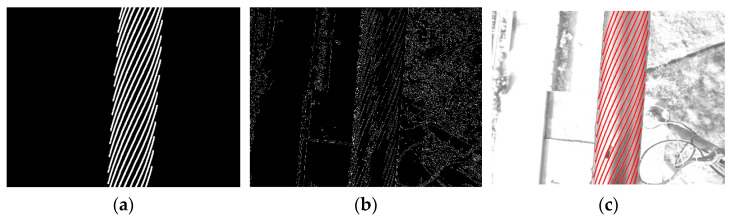
The result of strand segmentation by the proposed method. (**a**) Segmentation template; (**b**) Edge detection result with FoGDbED; (**c**) Strand segmentation by the proposed method.

The effect of strand segmentation and correction is shown in Figure 8. Figure 8a shows the steel wire strand segmentation template. In order to facilitate viewing, the grayscale with the median value of 1 in the template is replaced with 255. Figure 8b shows the edge information extracted by FoGDbED from the surface image of steel wire rope with defects. Figure 8c shows the results of strand cutting by this method. In order to better show the segmentation effect, in Figure 8c, the best matching template area is marked in red. The results show that the method in this paper can simply and directly segment the steel wire rope strands.

### 2.3. Creation of Spatiotemporal Gray Sample Set

In view of the structural particularity of steel wire rope, the image representation of different positions of normal steel wire rope is similar. The similarity is mainly reflected in the images of the upper surface of steel wire rope at different positions collected in real-time under outdoor natural light. If the same position in the image is the edge of rope strand, rope strand surface, or oil sludge surface, the gray value is similar; The difference mainly occurs in the case of gray mutation, and the mutation area is usually shown as a fracture defect.

**Figure 9:**
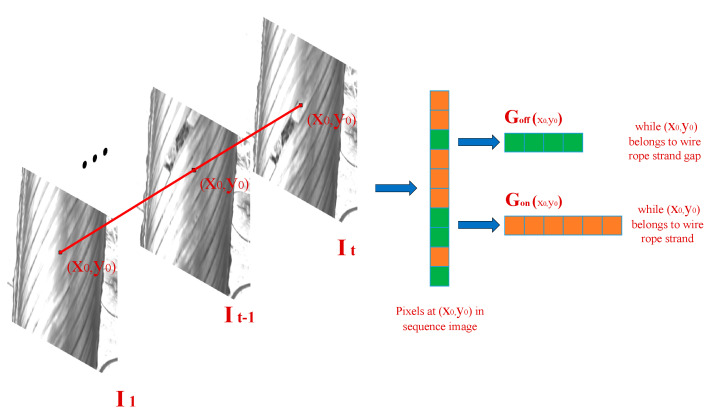
Composition of the temporal gray sample set.

Classical background modeling methods such as VIBE, KNN, and MOG2 will appear in a large number of foreground regions when processing images with rapidly changing texture, which cannot be directly used for wire rope surface defect segmentation. In this paper, a new temporal and spatial gray sample set is constructed to solve the problem of defect detection in the case of rapid texture change. Inspired by the dynamic background modeling method and combined with the gray change characteristics of wire rope surface image, this paper constructs the gray sample set in the space-time domain for the first time when the camera moves relative to the scene. The wire rope defect detection process is divided into dynamic wire rope background model construction and foreground defect detection process. The dynamic wire rope background model is realized by building a grayscale sample set in the space-time domain, and the detailed construction process is as follows:

Considering the sequence of pixel grayscale values of the same point in the time domain as a process of pixel grayscale changes with time, for the point $\left(x_{0},y_{0}\right)$, the set of pixel grayscale history values G in time t is

(9) $$G\left(x_{0},y_{0}\right)=\left\{g_{1},\ldots ,g_{t}\right\}$$

where $1\leq i\leq t\;\mathrm{and}\;g_{1},\ldots ,g_{t}$ are the grayscale values of the corresponding images at $\left(x_{0},y_{0}\right)$ from moment 1 to moment t. In practical application, the pixels in set $\left\{g_{1},\ldots ,g_{t}\right\}$ may be in different positions at different times, and may belong to the rope strand edge or rope strand surface. The average gray level of the pixels at the rope strand edge is lower than the average gray level of the pixels on the rope strand surface. If they are not distinguished, the gray level estimation value will be in an unstable state, affecting the accuracy of defective pixel segmentation. Therefore, the composition of the sequential gray sample set of the wire rope image sequence is shown in Figure 9. Continuously take the gray value of the pixels at the same position as the t-frame images. If the point is on the rope strand, the set is recorded as $G_{on}\left(x_{0},y_{0}\right)$; If the point is in the edge area of the rope strand, the set is recorded as $G_{off}\left(x_{0},y_{0}\right)$, and the sequential gray sample set is $G_{on}\left(x_{0},y_{0}\right)\cup \;G_{off}\left(x_{0},y_{0}\right)$.

**Figure 10:**
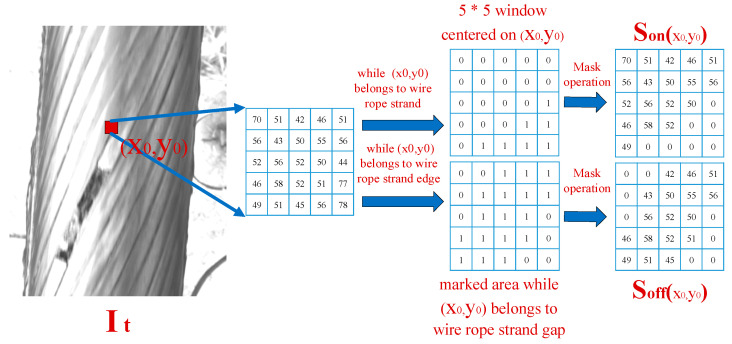
Composition of the spatial gray sample set.

In the spatial domain of the current wire rope image, combined with the structural characteristics of the sealed wire rope, if the pixels at position $\left(x_{0},y_{0}\right)$ and the adjacent pixels in the wire rope surface image are the same rope strand or the same rope strand, the gray values of the edge are similar. The spatial gray level set of pixel points is shown in Figure 10. Take point $\left(x_{0},y_{0}\right)$ as the 5 ∗ 5 area of the midpoint, and take the same area in the mask image obtained after rope strand segmentation. If the pixel is located in the rope strand area (i.e., the corresponding value in the mask image obtained by rope strand segmentation is 0), take all pixel gray level value sets of 0 in the 5 ∗ 5 pixel area as $S_{on}\left(x_{0},y_{0}\right)$; If the pixel is located in the rope strand edge area (that is, the corresponding value in the mask image obtained by rope strand segmentation is 1), take the gray value set of all pixels with 1 in the 5 ∗ 5 pixel area as $S_{off}\left(x_{0},y_{0}\right)$; Finally, the spatial gray sample set is $S_{on}(x_{0},y_{0})\cup \;S_{off}\left(x_{0},y_{0}\right)$.

The spatiotemporal gray sample set constructed in this paper can be expressed as two subsets:

(10) $$M_{on}\left(x_{0},y_{0}\right)=S_{on}\left(x_{0},y_{0}\right)\cup \;G_{on}\left(x_{0},y_{0}\right)$$

(11) $$M_{off}\left(x_{0},y_{0}\right)=S_{off}\left(x_{0},y_{0}\right)\cup \;G_{off}\left(x_{0},y_{0}\right)\;$$

where $M_{on}\left(x_{0},y_{0}\right)$ refers to the spatiotemporal gray sample set at $\left(x_{0},y_{0}\right)$ on the rope strand, and $M_{off}\left(x_{0},y_{0}\right)$ refers to the spatiotemporal gray sample set at $\left(x_{0},y_{0}\right)$ on the edge of the rope strand. The spatiotemporal gray sample set constructed in this paper has good adaptability to gradual illumination, uneven illumination, reflective shadows, oil sludge, and so on.

### 2.4. Wire Rope Defect Detection

In practical application, the starting point of wire rope detection can be intervened manually to ensure that there is no fracture in the initialization stage of the spatiotemporal sample set. When the model is initialized, the t-frame images are used for spatiotemporal gray modeling. When t is set appropriately, a stable dynamic spatiotemporal gray sample set can be obtained.

The principle of pixel gray replacement in the spatiotemporal gray sample set is to calculate the gradient value between the current pixel value and the pixel mean value in the spatiotemporal sample set. If the gradient value is less than the threshold $T_{0}$ ($T_{0}$ is the empirical threshold), it is determined that the current pixel is updated into the sample set, otherwise, it is not updated because the larger the pixel value gradient, it indicates that the pixel may be a defective pixel and should not be updated into the background. According to the principle of time-space first out of the queue, the current sample set updating principle will not introduce defective pixels due to the gradual illumination between image frames, uneven illumination inside the image, reflective shadow, oil sludge, and other conditions in the natural environment, so it has good adaptability to the complex environment.

The spatiotemporal gray sample set is used to detect defects in the process of traversal each pixel in the current image. Take $\left(x_{0},y_{0}\right)$ as an example to calculate the sample value distance in the sample set with its corresponding sample number W. To calculate the distance, we need to select the sub-set of the rope strand $\left\{S_{on}\left(x_{0},y_{0}\right)\cup \;G_{on}\left(x_{0},y_{0}\right)\;\right\}$ or the sub-set of the rope strand edge $\left\{S_{off}\left(x_{0},y_{0}\right)\cup \;G_{off}\left(x_{0},y_{0}\right)\right\}$ according to the pixel location, determine whether the distance is less than the corresponding set threshold $T_{on}$ (rope strand pixel threshold) or $T_{off}$ (rope strand edge pixel threshold), and count the number of samples less than the set threshold E.

Then, the proportion of similar samples $Q\left(x_{0},y_{0}\right)$ is:

(12) $$Q\left(x_{0},y_{0}\right)=\frac{E}{V}$$

where V is the number of the sample set, E is the number satisfied with the distance constraints of $T_{on}$ or $T_{off}$. When $Q\left(x_{0},y_{0}\right)\geq T_{1}$ and $T_{1}$ is the empirical threshold, the current pixel $\left(x_{0},y_{0}\right)$ is the pixel of the background; when $Q\left(x_{0},y_{0}\right)<T_{1}$, the current pixel $\left(x_{0},y_{0}\right)$ may be the pixel of the defect. If the gray value of the current pixel is greater than the average gray value of its corresponding sample set and exceeds the threshold $T_{2}$ (empirical threshold), the current pixel $\left(x_{0},y_{0}\right)$ is determined to be an anomalous pixel. However, the pixel does not belong to the defect area, because the average of a defective pixel is smaller than that of surrounding pixels and the current pixel may be a pixel of the strong reflection region.

## 3. Algorithm Overview

**Figure 11:**
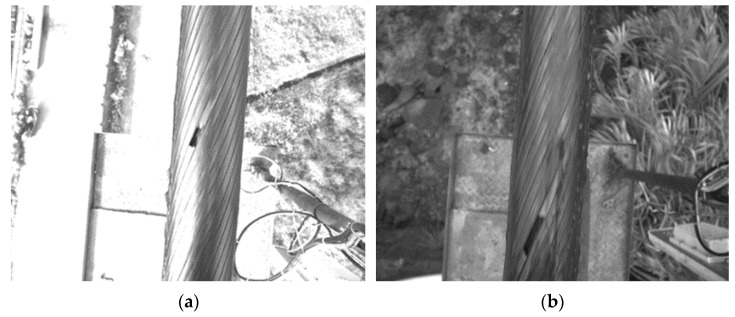
Wire rope image with a defect. (**a**) Strong light; (**b**) Weak light.

The defect of the sealing steel wire rope is mainly reflected in the fracture of the surface rope strand. Therefore, the detection of the surface defect of the steel wire rope shall be based on the rope strand detection. As shown in Figure 11a,b, the fracture defect must be connected with at least one pair of adjacent rope strand edges under strong or weak light.

Steel wire rope surface defect detection method based on segmentation template and spatiotemporal gray sample set (STSGSS) is shown in the algorithm in Algorithm 2. The steel wire rope segmentation template is created in advance, and the problem of rope strand segmentation in real-time images in complex environments is solved. On this basis, continuous t-frame images are collected to build a spatiotemporal gray sample set, the content of the sample set is updated in real-time, the dynamic background modeling of the steel wire rope surface is realized, the distance between each pixel in the current image and its sample set is calculated, and the foreground defect area on the surface of steel wire rope is detected.
**Algorithm 2.** Steel wire rope surface defect detection method based on segmentation template and spatiotemporal gray sample set.**Inputs:** Sequence images, time-domain frames P **Output:** Sequence Images D(x,y)s containing defects 1: get ROI mask with Formula (1) and Formula (2) 2: t=0 3: **while** sequence images are not empty 4:  get ROI of the real-time image with ROI mask 5:   get N(x,y) with RSCM (algorithm 1) 6:    t=t+1 7:   **if** t≤P 8:      use queues save $S_{off}\left(x,y\right),G_{off}\left(x,y\right)\;,S_{on}\left(x,y\right),G_{on}\left(x,y\right)$ 9:   **else** 10:      get gray AVG of $S_{off}\left(x,y\right)\cup^{\;}G_{off}\left(x,y\right)\;\;\mathrm{or}\;S_{on}\left(x,y\right)\cup^{\;}G_{on}\left(x,y\right)$ 11:      **if** the gradient between AVG and the current pixel $\leq T_{0}$ 12:       update $S_{off}\left(x,y\right)\cup^{\;}G_{off}\left(x,y\right)\;\;\mathrm{or}\;S_{on}\left(x,y\right)\cup^{\;}G_{on}\left(x,y\right)$ by rule of          FIFO (first in first out) 13:     **end if** 14:     get Q(x,y) with Formula (12) 15:     **if** $Q\left(x,y\right)\leq T_{1}$ and $Q\left(x,y\right)\leq T_{2}$ 16:      D(x,y)=255 17:    **else** 18:      D(x,y)=0 19:     **end if** 20:    open operation of the morphological method for D(x,y) 21:    analyse D(x,y) connected domain and remove small connected regions 22: **end while** 23: return D(x,y)s

## 4. Experimental Validation

### 4.1. Experimental Environment Construction

**Figure 12:**
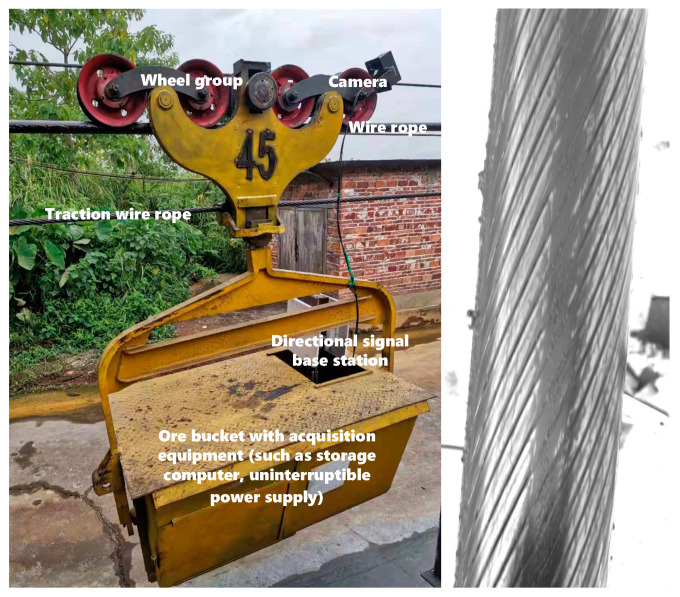
Steel wire image acquisition system.

We considered the heavy load rope on a lead-zinc mine ropeway system as the experimental object. The load rope uses two layers of Z-sealed steel wire rope. The diameter is 50 mm, the length is 1422 m, and the load rope is used to carry a full load in a mine bucket car. In this study, dedicated image acquisition and a simultaneous transmission system were built using an empty bucket truck, as shown in Figure 12. The bucket truck contained an industrial control computer, two-directional base stations, a camera power supply, a gigabit Ethernet switch, and an uninterruptible power supply (UPS). The image acquisition device was fixed to the axle bearing of the bucket truck, with the camera axis coinciding with the rope. The image acquisition part of the system comprised an MV-CA030-10GC industrial camera model (Hikvision, Hangzhou, Zhejiang, China). The camera supports the GIGE protocol and has a resolution of 1920 × 1440 pixels, a bit depth of 8 bits, and a real-time image acquisition frame rate of 20 frames/s. The bucket truck travels at a speed of 0.2 m/s. The two-directional base stations in the bucket truck and those installed at both ends of the ropeway form a wireless communication network. For real-time image preview and fracture analysis, the industrial camera collects images, which the industrial control computer in the bucket transmits to the wire rope surface defect analysis host in a monitoring room through the wireless network built between the base stations.

### 4.2. Experimental Comparison and Analysis

The principle of the method proposed in this paper is to establish a dynamic spatiotemporal sample set for each pixel in the image and use this sample set to realize the background modeling of the wire rope surface image. Classical dynamic background modeling methods such as VIBE, KNN, and MOG2 are used to construct dynamical models of every pixel in the image. Therefore, in the comparative experimental design, VIBE, KNN, and MOG2 are used to replace the spatiotemporal gray sample set based on the segmentation template and other algorithm steps are consistent.

Because there is little data on the surface defects of steel wire rope, in order to intuitively describe the effect of the algorithm, the quantitative index false detection rate (*FAR*) is selected to evaluate the performance of each algorithm in the experiment. The expression of *FAR* is:

(13) $$FAR=\frac{FP}{BP}\times 100\%$$

where *FP* is the number of pixels with false detection as the foreground, and *BP* is the total number of pixels in the wire rope area.

**Figure 13:**
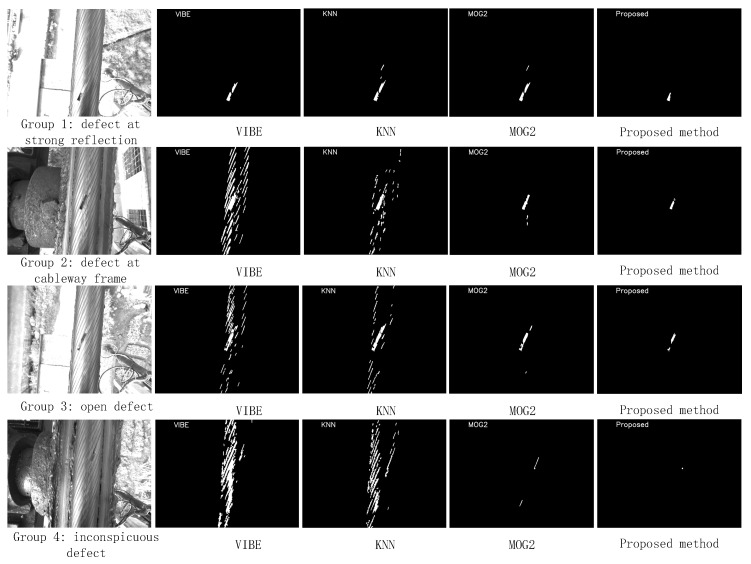
Detection effects of VIBE, KNN, and MOG2 and the proposed method for fracture.

In order to verify the defect detection effect of this algorithm in different environments, for the first group of experiments, four consecutive image sequences containing structural breaks for experimental comparison were selected; each image sequence contained 300 frames and one defect. The detection effect at the defect is shown in Figure 13, and a comparison of detection effects is shown in Table 1.

In the first group of tests, as shown in Figure 13, the four methods of defect area are detected, but it can be seen from Table 1 that the error detection rate of VIBE is greater than that of KNN, and the error detection rate of KNN is greater than that of MOG2. The error detection rate of the proposed method is much lower than other methods. In the first group of experiments, the main reason for the poor effect of VIBE was that the model took each pixel as the center, R being the radius of the region, to obtain neighboring pixel information. The pixel information in this part and in the time series image was not distinguished between the rope strand and the rope strand gap, and the model random turnover data mechanism was not applicable to the current detection environment. This resulted in a large area of rope strand gap pixels in the image being mistakenly considered defective areas.

The KNN algorithm principle does not contain image space information, and the difference between the rope strand and the rope strand gap is not considered in the timing information, so some rope strand gap pixels were mistakenly considered defective areas.

MOG2 model the temporal pixel information with a hybrid Gaussian model, which has a low false detection rate when multiple distribution parameters are used but is time-consuming. The proposed algorithm is highly adaptable, and no false detection occurred for the above common cases, as shown in Figure 13, which shows the detection effect of the first group and the third group.

VIBE, KNN, and MOG2 had high false detection rates for bright reflections, and our algorithm did not use false detection. Under strong light, VIBE, KNN, and MOG2 had lower false detection rates on the rope strand gap because the difference between the rope strand gap and the rope strand grayscale level is small under strong light.

**Figure 14:**
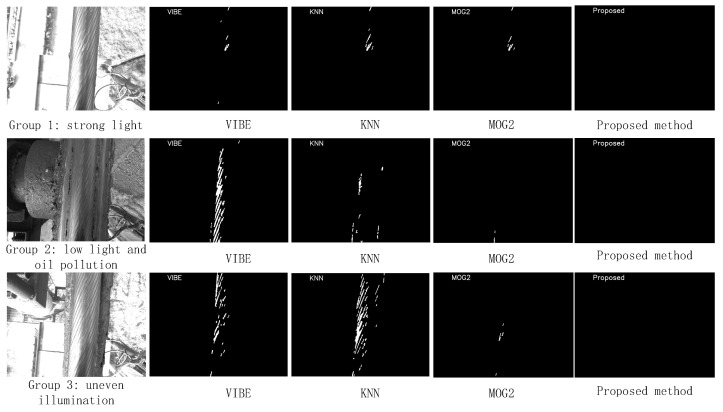
Detection effects of VIBE, KNN, and MOG2 and the proposed method.

For the second group of experiments, three consecutive image sequences involving special circumstances, such as strong light, serious oil stains out of the ropeway frame, and uneven light, were selected for comparison. Each image sequence contained 300 frames. The first group of experiments paid attention to the defect detection effect, and the second group of experiments paid attention to the influence of different detection environments on the algorithm. The detection effect is shown in Figure 14, and the false detection rate FAR of the algorithms in the special environment is shown in Table 2.

For the second group of tests, as shown in Figure 14, the complex environment has varying degrees of impact on VIBE, KNN, and MOG2. It can be seen from Table 2 that the complex environment has little impact on the method in this paper, and the false detection area is mainly reflected in the oil stain with a dark grayscale. The second group of experiments showed that bright light has little effect on VIBE, KNN, and MOG2 and basically has no effect on the algorithm used in this study. However, the bright reflective area on the rope strand was still mistakenly detected as a defective area by VIBE, KNN, and MOG2. Weak light oil has a greater impact on VIBE because it does not consider the sealed wire rope texture and its random turnover mechanism is unsuitable for this environment. KNN and MOG2 algorithms are weakly affected by oil stains, but oil stains’ heavy local highlighted pixels are misdetected. In the case of uneven illumination, the pixel grayscale difference between the rope strand gap and the rope strand has a greater impact on the VIBE and KNN algorithms, mainly because VIBE misapplies spatial information and VIBE and KNN are not applicable to fast, regular-texture-change image sequences. The multi-distribution model in MOG2 simulates the grayscale distribution and fits the image sequence of regular texture changes to a certain extent, so it is less affected by regular texture changes on the surface of the sealed wire rope. The algorithm in this study is designed to apply the spatial texture distribution law of a wire rope from two perspectives, time domain and space domain, and the pixels with gradient mutation are treated separately in the model-updating strategy, so the proposed algorithm has good adaptability to the complex environment in sealed wire rope detection.

## 5. Conclusions

In this study, we proposed a wire rope surface defect detection method based on a segmentation template and spatiotemporal gray sample set in a complex environment. Different from previous work, the proposed method has strong adaptability to the surface defect detection of sealed steel wire ropes, and its principles can be extended to other types of sealed steel wire rope defect detection work. In addition, as it has good adaptability, this method does not require a large number of samples of defects arising from lubricating oil, adhering dust, natural light, metal or oil reflection, and other types of complex environments. It can be directly used for wire rope strand division and fracture detection. In this study, we constructed a steel wire rope segmentation template, and the position of the rope strand in a real-time image could be marked and segmented by calculating the best overlap response value after shift operation along the rope strand direction. In the relative motion environment between the camera and the scene, the gray sample set in the space-time domain was constructed for the first time to update the dynamic background of the wire rope surface in real-time, so as to realize the robust detection of wire rope surface defects in complex environments such as uneven light, strong light, weak light, and reflection. By constructing a real-world experimental environment for wire rope defect detection, collecting real data to verify the effectiveness of the proposed method, and comparing the results with those of classical background modeling methods VIBE, KNN, and MOG2, the proposed method was verified to have better performance in sealed wire rope defect detection applications, and it was more accurate and more adaptable to a complex environment.

## Figures and Tables

**Table 1:** FAR statistical results of VIBE, KNN, and MOG2 and the proposed method at different groups.

Experiments	VIBE	KNN	MOG2	Proposed Method
Group 1	4.35%	3.62%	1.17%	0.02%
Group 2	5.82%	4.64%	1.89%	0.05%
Group 3	4.87%	4.23%	1.42%	0.03%
Group 4	5.76%	4.92%	2.04%	0.09%

**Table 2:** FAR of VIBE, KNN, MOG2, and the proposed method in the complex environment.

Experiments	VIBE	KNN	MOG2	Proposed Method
Strong light	4.12%	3.21%	1.02%	0.02%
Weak light and oil stain	6.17%	3.21%	0.74%	0.11%
Uneven light	5.04%	6.73%	0.69%	0.03%
